# Prevalence and 3-month follow-up of cerebrovascular MRI markers in hospitalized COVID-19 patients: the CORONIS study

**DOI:** 10.1007/s00234-024-03411-1

**Published:** 2024-07-02

**Authors:** Theresa J. van Lith, Wouter M. Sluis, Naomi T. Wijers, Frederick J.A. Meijer, Karin Kamphuis-van Ulzen, Jeroen de Bresser, Jan Willem Dankbaar, Quirijn de Mast, Frederikus A. Klok, Suzanne C. Cannegieter, Marieke J. H. Wermer, Menno V. Huisman, Anil M. Tuladhar, H. Bart van der Worp, Frank-Erik de Leeuw

**Affiliations:** 1 Department of Neurology, Donders Center for Medical Neuroscience, Radboud University Medical Center, PO Box 9101, Nijmegen, 6500 HB the Netherlands; 2 Department of Neurology and Neurosurgery, Brain Center, University Medical Center Utrecht, Utrecht, the Netherlands; 3 Department of Neurology, Leiden University Medical Center, Leiden, The Netherlands; 4 Department of Medical Imaging, Radboud University Medical Center, Nijmegen, The Netherlands; 5 Department of Radiology, Leiden University Medical Center, Leiden, the Netherlands; 6 Department of Radiology and Nuclear Medicine, University Medical Center, Utrecht University, Utrecht, The Netherlands; 7 Department of Internal Medicine, Radboud Center for Infectious Diseases, Radboud University Medical Center, Nijmegen, The Netherlands; 8 Department of Medicine - Thrombosis and Hemostasis, Leiden University Medical Centre, Leiden, The Netherlands; 9 Department of Clinical Epidemiology, Leiden University Medical Centre, Leiden, The Netherlands; 10 Department of Neurology, University Medical Center, Groningen, The Netherlands

**Keywords:** COVID-19, MRI, Cerebrovascular disorders, Brain ischemia, WMH, SARS-CoV-2

## Abstract

**Purpose:**

To investigate the prevalence of cerebrovascular MRI markers in unselected patients hospitalized for COVID-19 (Coronavirus disease 2019), we compared these with healthy controls without previous SARS-CoV-2 infection or hospitalization and subsequently, investigated longitudinal (incidental) lesions in patients after three months.

**Methods:**

CORONIS (CORONavirus and Ischemic Stroke) was an observational cohort study in adult hospitalized patients for COVID-19 and controls without COVID-19, conducted between April 2021 and September 2022. Brain MRI was performed shortly after discharge and after 3 months. Outcomes included recent ischemic (DWI-positive) lesions, previous infarction, microbleeds, white matter hyperintensities (WMH) and intracerebral hemorrhage and were analysed with logistic regression to adjust for confounders.

**Results:**

125 patients with COVID-19 and 47 controls underwent brain MRI a median of 41.5 days after symptom onset. DWI-positive lesions were found in one patient (1%) and in one (2%) control, both clinically silent. WMH were more prevalent in patients (78%) than in controls (62%) (adjusted OR: 2.95 [95% CI: 1.07–8.57]), other cerebrovascular MRI markers did not differ. Prevalence of markers in ICU vs. non-ICU patients was similar. After three months, five patients (5%) had new cerebrovascular lesions, including DWI-positive lesions (1 patient, 1.0%), cerebral infarction (2 patients, 2.0%) and microbleeds (3 patients, 3.1%).

**Conclusion:**

Overall, we found no higher prevalence of cerebrovascular markers in unselected hospitalized COVID-19 patients compared to controls. The few incident DWI-lesions were most likely to be explained by risk-factors of small vessel disease. In the general hospitalized COVID-19 population, COVID-19 shows limited impact on cerebrovascular MRI markers shortly after hospitalization.

**Supplementary Information:**

The online version contains supplementary material available at 10.1007/s00234-024-03411-1.

## Introduction

Infection with severe acute respiratory syndrome coronavirus 2 (SARS-CoV2) is associated with both venous and arterial thrombo-embolic events including ischemic stroke [1–3]. Pulmonary embolism is the most frequent thrombo-embolic complication, but ischemic stroke was also frequently reported in patients with Coronavirus disease 2019 (COVID-19) ranging from 0.9 to 2.0% [1, 4–8]. This was a higher incidence of ischemic stroke compared to hospitalized patients with influenza (0.2–0.9%). An even higher incidence (up to 2.7%) was reported in critically ill COVID-19 patients admitted to an intensive care unit (ICU) [9–11].

Other MRI markers of cerebrovascular disease such as microbleeds, intracerebral hemorrhage and intracranial vessel wall enhancement have also been found in retrospective cohorts of patients with COVID-19 [9, 12–15]. The procoagulant and proinflammatory response of COVID-19 may result in ‘clinically silent’ or ‘covert’ ischemic lesions and other cerebrovascular MRI abnormalities during the disease course, in addition to symptomatic ‘overt’ ischemic stroke (associated with clear neurological deficits).

Brain imaging in previous studies in patients with COVID-19 was often performed in selected critically-ill patients, presenting with overt neurological symptoms with a clinical indication for imaging. The prevalence of cerebrovascular lesions on MRI without overt symptoms in unselected patients admitted with COVID-19 remains unknown and this has never been compared to controls from the general population with proven absence of COVID-19. In addition, in none of the previous studies follow-up imaging weeks to months after infection to detect incident subacute cerebrovascular changes has been performed.

Therefore, we investigated prevalence and 3-month incidence of asymptomatic (silent) cerebral ischemia and other cerebrovascular MRI markers in the CORONavirus and Ischemic Stroke (CORONIS) study, a prospective cohort of unselected hospitalized patients with COVID-19. To evaluate the effect of severe COVID-19 (requiring hospitalization) on cerebrovascular MRI markers, we compared this prevalence with healthy controls without proven infection and without hospitalization.

## Methods

### Study design

This study is part of the CORONIS study, a prospective observational cohort study that investigates the prevalence, risk factors and long-term effects of (silent) MRI markers of cerebrovascular disease in hospitalized patients with COVID-19. The study protocol has been published previously [16]. The Medical Review Ethics Committee region Arnhem-Nijmegen approved the study (NL75780.091.20). All patients provided written informed consent.

### Study population

All adult patients admitted between April 2021 and September 2022 to one of three Dutch academic hospitals with laboratory-confirmed COVID-19 infection, with or without clinically overt ischemic stroke during admission, regardless of any clinical (neurological) symptoms were eligible for inclusion. For the present analysis, we excluded patients with COVID-19 and ischemic stroke or a transient ischemic attack (TIA) during admission as the primary aim of this study was to detect cerebrovascular MRI markers in patients with COVID-19 without clinically overt stroke. Exclusion criteria included contraindications to MRI or intravenous gadolinium, pregnancy, life expectancy shorter than three months, major disease interfering with study participation or follow-up or inability to provide informed consent. Controls from the general population with a clinically and laboratory proven absence of COVID-19 infection, matched on age and sex), were recruited among the patients’ next of kin and social environment. We matched the controls at an approximately 1:3 ratio with the patients, as we considered this number sufficient for exploratory analyses. Since no information from brain MRIs in COVID-19 patients was yet available, we decided to focus mostly on this group, hence the 1:3 ratio. Because recruitment of controls without a history of COVID-19 infection slowed down during the pandemic, we did not reach the target of 60 included controls as described in the study protocol. To ensure the controls were COVID-19 negative, all controls were asked whether they had COVID-19 during the pandemic, or if they had COVID-19 related symptoms but refrained from testing. Additionally, we performed an Anti-Sars-CoV-2 nucleocapsid laboratory test to exclude subjects with a previous asymptomatic COVID-19 infection. In unvaccinated controls, an additional Anti-Sars-CoV-2 spike test was performed.

### Data collection

We collected information on demographics, comorbidities, medication use and vaccination status at baseline, which was the moment of enrolment in the study (for patients during admission or shortly after discharge). This included information on cardiovascular risk factors e.g. hypertension, diabetes mellitus, smoking, hypercholesterolemia, atrial fibrillation and a previous TIA or ischemic stroke. For patients with COVID-19, laboratory test results and clinical data (e.g. hospital stay in days, complications, mechanical ventilation need, medication) during admission were collected.

### Brain MRI analysis

At baseline, patients and controls underwent 3-Tesla brain MRI including T1-weighted imaging, 2D or 3D fluid-attenuated inversion recovery (FLAIR), diffusion-weighted imaging (DWI) including an apparent diffusion coefficient (ADC) and susceptibility-weighted imaging (SWI). Details of the scanning protocol in each participating center are shown in supplemental Table 1. To investigate the dynamics of silent ischemia in patients with COVID-19, they were asked to undergo follow-up MRI three months after baseline MRI (with the same scanning protocol). The controls did not undergo follow-up MRI as we did not expect incident asymptomatic cerebral ischemia over a three months course in these individuals.

### MRI markers of cerebrovascular disease

The standardized evaluation protocol consisted of specific cerebrovascular MRI markers of interest, described in supplemental Table 2. Markers of cerebral small vessel disease were rated according to the Standards for Reporting Vascular Changes on nEuroimaging (STRIVE-2) criteria [17, 18]. An acute ischemic lesion (incidental DWI-positive lesion) was defined by the presence of diffusion restriction on DWI. Cerebral microbleeds were defined as small (2–10 mm) round areas of signal void with blooming seen on gradient-echo sequences and rated according to the microbleed anatomical rating scale, those with a larger diameter were referred to as an intracerebral hemorrhage [19].

All brain MRIs were anonymized and evaluated by one of four experienced neuroradiologists (FM, KKvU, JdB and JWD) using a standardized, structured protocol. Because of considerable variations in SWI sequences among the different centers and to ensure the coherence of the assessments, one experienced rater (TJvL) evaluated all SWI scans for microbleeds. In cases where uncertainty arose, these assessments were subjected to review and confirmation by an experienced neurologist (FEdL).

### Outcome

The primary objectives were to conduct a cross-sectional comparison of the prevalence of MRI markers of cerebrovascular disease in patients with COVID-19 compared with controls at baseline. Second, our aim was to investigate longitudinal changes (incident cerebrovascular lesions) over time (three months) in patients hospitalized for COVID-19.

### Statistical analysis

Frequencies and counts were described for categorical data (n, %). For quantitative data we reported means with standard deviation (SD) and medians with interquartile range (IQR). Demographics, medical history and brain MRI markers were compared between groups (COVID-19 vs. controls and ICU vs. non-ICU) with Student’s t-test or chi-square test as appropriate with corresponding 95% confidenceintervals (95% CI) for (difference in) proportions. Missing values were not imputed. Uni- and multivariable (ordinal) logistic regression was used to analyze the relationship between COVID-19 infection and the primary and secondary outcomes displayed as crude odds ratios (OR) and adjusted odds ratios (aOR) with corresponding 95% CIs adjusted for age, sex, hypertension, diabetes mellitus, hypercholesterolemia, BMI, smoking behaviour and study site. *P*-values less than 0.05 were considered statistically significant. All analyses were performed with R, version 4.2.2. We reported this article in accordance with the STROBE (Strengthening the Reporting of Observational Studies in Epidemiology) guidelines (Supplemental Material) [20].

## Results

### Study population

**Fig. 1:**
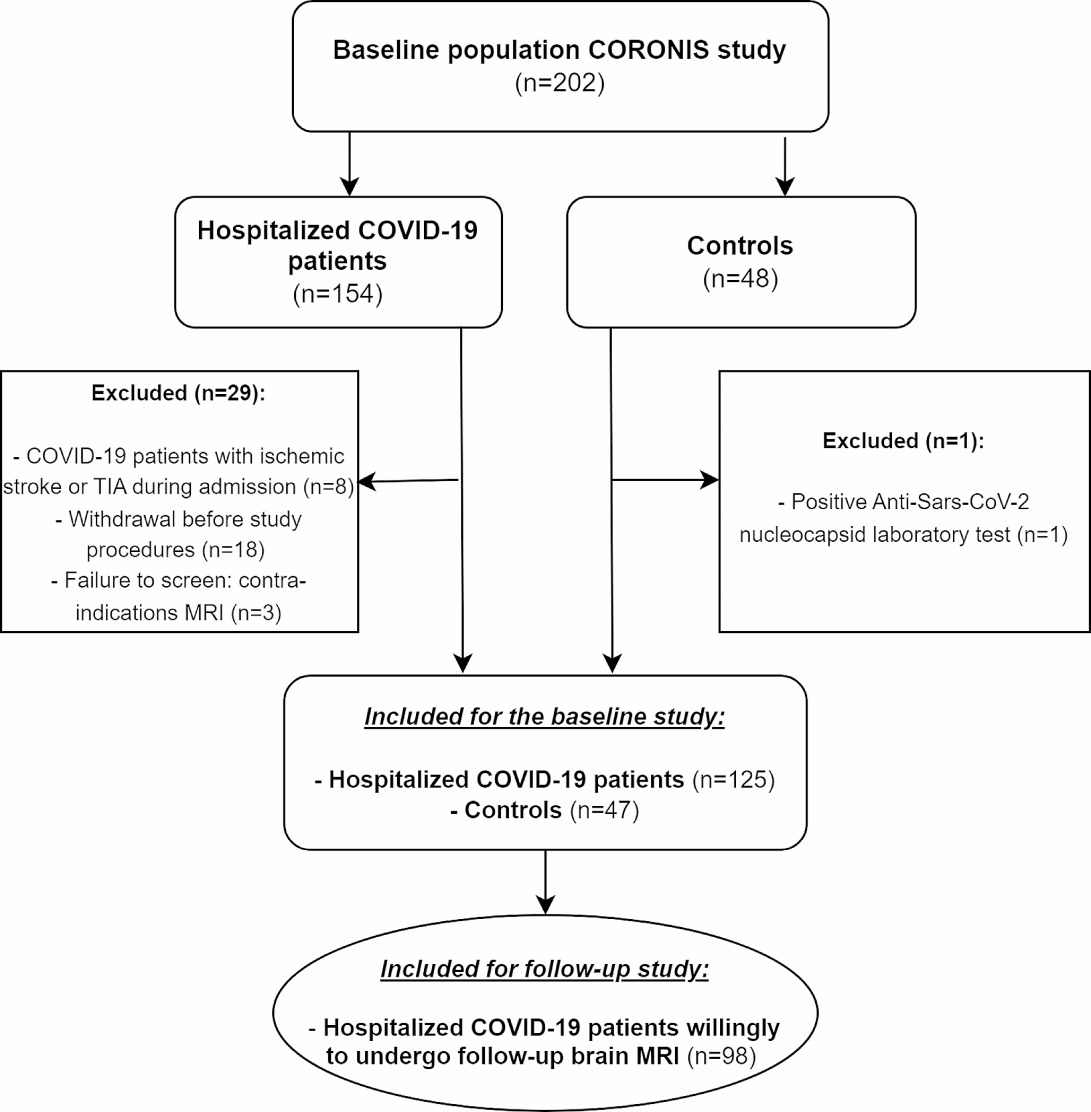
Flowchart of inclusion

In total, 154 patients with COVID-19 and 48 controls were enrolled in CORONIS. After exclusion of participants who withdrew their consent before MRI was performed (*n* = 18), patients with a contra-indication for MRI (*n* = 3), patients with a symptomatic stroke or TIA during admission (*n* = 8), and controls with a positive Anti-Sars-CoV-2 nucleocapsid test (*n* = 1), 125 patients with COVID-19 and 47 controls were analyzed (Fig. 1).

### Patient characteristics

Patients with COVID-19 were comparable to controls regarding age and sex, with a mean age of 58 (SD: 12.8) years and 40.0% (50/125) were female after frequency matching during inclusion. There was a difference in vaccination rate between patients and controls (37.9% vs. 91.7%, *p = 0.001)* (Table 1). Additional clinical information is presented in supplemental Table 3. Median time between positive PCR in patients and inclusion was 16.0 days [IQR: 5.0–12.0].

**Table 1:** Baseline characteristics COVID-19 patients vs. controls

	COVID-19 +	Controls	*P*-value
Total number of patients (n)	125	47	
Female, n (%)	50 (40.0)	22 (46.8)	0.420
Age at inclusion, years (mean, (SD))	58.1 (12.8)	60.7 (12.6)	0.235
BMI, kg/m2 (median [IQR])	27.8 [24.8, 32.1]	26.73[23.6, 28.5]	***0.024***
Vaccinated before admission/inclusion, n (%)	47 (37.9)	11 (91.7)	***< 0.001***
Race, n (%)			0.193
Caucasian/white	103 (84.4)	43 (91.5)	
Black	3 (2.5)	0 (0.0)	
North-African	10 (8.2)	1 (2.1)	
Hispanic	2 (1.6)	0 (0.0)	
Asian	2 (1.6)	0 (0.0)	
Mixed	2 (1.6)	3 (6.4)	
*Medical history*			
Previous or current smoker, n (%)	76 (60.8)	26 (55.3)	0.514
Previous or current alcohol use, n (%)	98 (78.4)	43 (91.5)	***0.047***
Diabetes Mellitus, n (%)	18 (14.4)	3 (6.4)	0.152
Hypertension, n (%)	48 (38.4)	7 (14.9)	***0.003***
Hypercholesterolemia, n (%)	46 (36.8)	8 (17.0)	***0.013***
Atrial fibrillation, n (%)	8 (6.4)	2 (4.3)	0.592
TIA, n (%)	5 (4.0)	0 (0.0)	0.164
Ischemic stroke, n (%)	5 (4.0)	0 (0.0)	0.164
Malignancy, n (%)	11 (8.8)	6 (12.8)	0.437
Venous thromboembolism (e.g. pulmonary embolism), n (%)	15 (12.0)	1 (2.1)	***0.047***
Pulmonary disease (e.g. COPD, asthma), n (%)	43 (34.4)	5 (10.6)	***0.002***
ICU admission, n (%)	27 (21.6)	N/A	N/A
Time of hospitalization, days (median [IQR])	8.0 [5.0–12.0]	N/A	N/A
Anticoagulant therapy during admission, n (%)	124 (99.2)	N/A	N/A
LMWH prophylactic dose	64 (51.2)	N/A	N/A
LMWH therapeutic dose	52 (41.6)	N/A	N/A
Other anticoagulant therapy (DOAC, vitamin K antagonist, factor Xa inhibitors)	8 (6.4)	N/A	N/A
Antiplatelet therapy during admission, n (%)	14 (11.2)	N/A	N/A
Pulmonary embolism during admission, n (%)	22 (17.6)	N/A	N/A

Abbreviations: COVID-19 = Coronavirus Disease 2019, BMI = Body Mass Index, TIA = transient ischemic stroke, COPD = chronic obstructive pulmonary disease, ICU = intensive care unit, DOAC = direct oral anticoagulation), N/A = not applicable

### Outcomes

#### Prevalence of MRI markers of cerebrovascular disease

At baseline, silent cerebral ischemia (DWI-positive lesions) was seen in one patient with COVID-19 (0.8%) and in one (2.1%) healthy control (1.3% difference; [95% CI; -5.7%, 3.1%]; *p* = 0.47). The MRI of the patient with COVID-19 showed multiple incidental DWI-positive lesions located in the white matter of the frontal and parietal lobe. This patient also had extensive segmental pulmonary embolism during hospital admission.

The prevalence of WMH was higher in patients (77.6%) compared to controls (61.7%) (15.9% difference; [95% CI, 0.2%, 531.6%]; *p* = 0.036). There was no difference in other MRI markers between cases and controls (Table 2**).** This was also the case when comparing patients admitted to an ICU with patients who were not (Supplemental Table 4). In one patient, as an incidental finding, an old asymptomatic cerebral venous sinus thrombosis (sigmoid sinus) was found that required no treatment.

**Table 2:** Cerebrovascular MRI markers in COVID-19 patients vs. controls

	COVID-19 +	Controls	*P*-value
Total number of participants, n	125	47	
Incidental DWI-positive lesions, n (%)	1 (0.8)	1 (2.1)	0.469
WMH, n (%)	97 (77.6)	29 (61.7)	***0.036***
WMH - Fazekas score, n (%)			
Fazekas 0 Fazekas 1 Fazekas 2 Fazekas 3	28 (22.4) 74 (59.2) 18 (14.4) 5 (4.0)	18 (38.3) 25 (53.2) 4 (8.5) 0 (0.0)	0.100
Previous cerebral infarction, n (%)	18 (14.4)	5 (10.6)	0.518
Delayed hypoxemia, n (%)	1 (0.8)	0 (0.0)	0.539
Cerebral hemorrhage, n (%)	6 (4.8)	3 (6.4)	0.678
Microbleeds, n (%)	29 (23.2)	6 (12.8)	0.130
*Location, n (%)*			0.460
Lobar	14 (48.3)	4 (66.7)	
Deep	6 (20.7)	0 (0.0)	
Cerebellar	2 (6.9)	0 (0.0)	
Lobar and deep	3 (10.3)	2 (33.3)	
Lobar and cerebellar	2 (6.9)	0 (0.0)	
Lobar, deep and cerebellar	2 (6.9)	0 (0.0)	
*Count, n (%)*			0.317
0	96 (76.8)	41 (87.2)	
1–10	24 (19.2)	5 (10.6)	
>10	5 (4.0)	1 (2.1)	

Abbreviations: COVID-19 = Coronavirus Disease 2019, DWI = diffusion-weighted imaging, WMH = white matter hyperintensities

#### New/incident MRI markers of cerebrovascular disease at 3 month follow-up

**Fig. 2:**
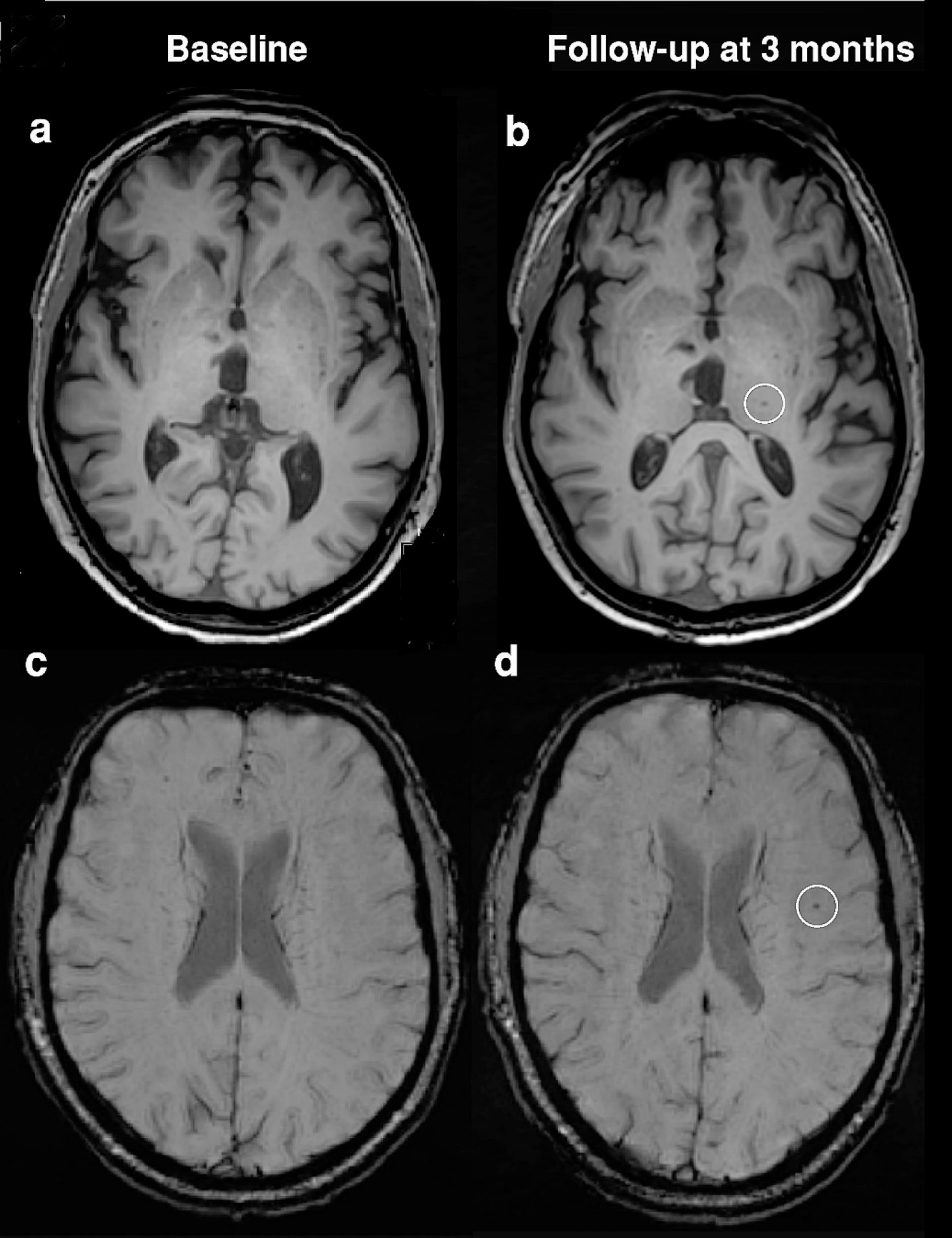
New cerebrovascular MRI markers in two COVID-19 patients; baseline MRI versus 3-month follow-up MRI. (**a**) Baseline T1 MRI scan patient 1 (**b**) Follow-up T1 MRI scan showing new cerebral infarction (oval) in patient 1 (**c**) Baseline SWI MRI scan in patient 2 (**d**) Follow-up SWI MRI scan showing new microbleed (white circle)) in patient 2. Abbreviations: MRI = magnetic resonance imaging, COVID-19 = Coronavirus Disease 2019, SWI = susceptibility weighted imaging

**Fig. 3:**
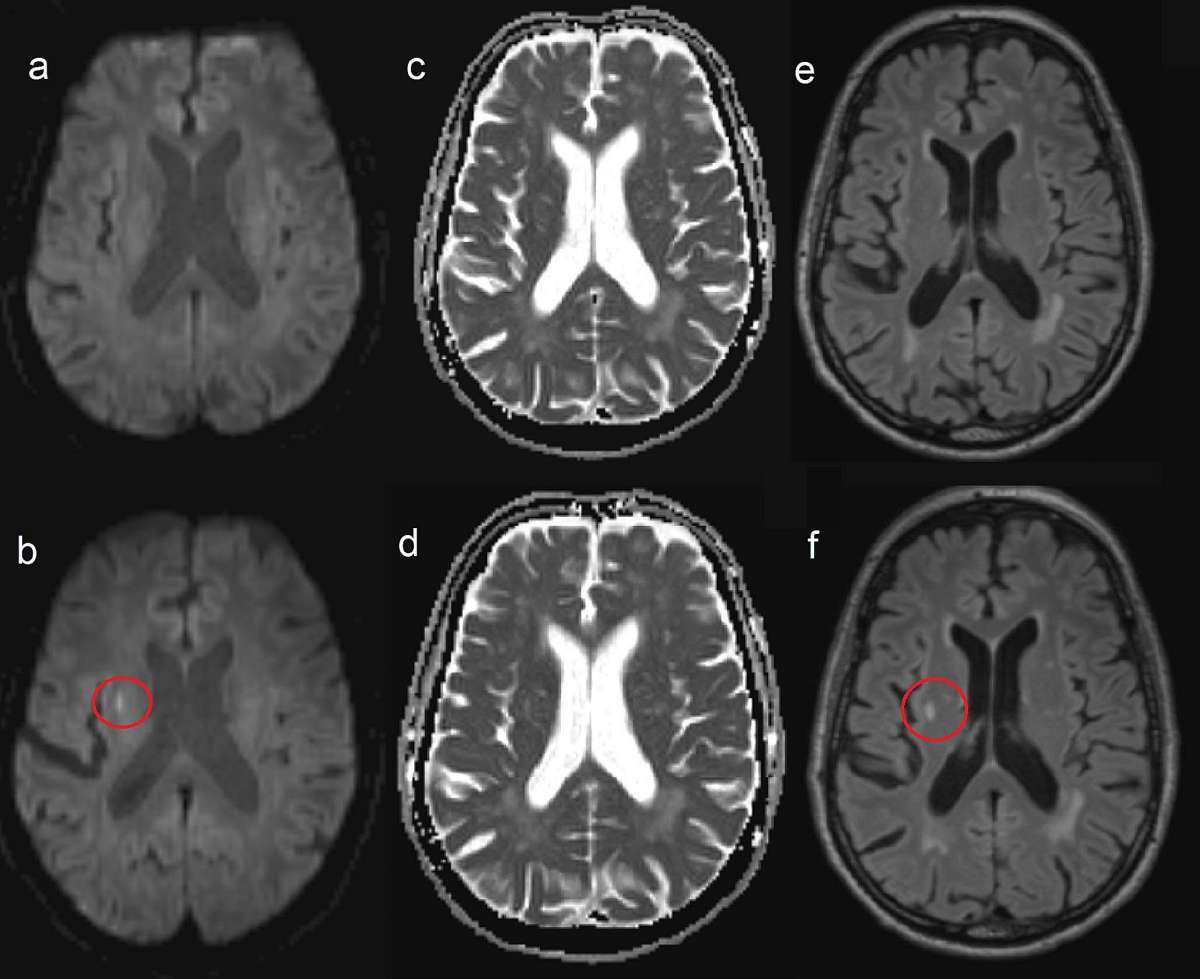
New cerebral infarction on 3-month follow-up MRI in 1 patient with COVID-19. (**a**) Baseline DWI-MRI scan in patient with COVID-19 (**b**) Follow-up DWI-MRI scan in same patient, with lacunar infarction (hyperintensity) right (red circle) (**c**) Baseline ADC-MRI scan in same patient (**d**) Follow-up ADC-MRI scan, no signs of recent ischemia (no corresponding hypointensity) (**e**) Baseline FLAIR MRI scan in same patient (**f**) Follow-up MRI showing corresponding hyperintense lesion in (**a**) indicating semi-recent lacunar infarction right, converted to WMH. Abbreviations: MRI = magnetic resonance imaging, COVID-19 = Coronavirus Disease 2019, DWI = diffusion-weighted imaging, ADC = apparent diffusion coefficient, FLAIR = fluid-attenuated inversion recovery, WMH = white matter hyperintensity

The subset of patients (*n* = 98, 78,4%) with a follow-up MRI had a mean age of 59.0 years (SD 12.3) and 37% were female. Five (5.1%) patients had new, silent cerebrovascular MRI markers after 3 months, including incidental DWI-positive lesions (1 patient, 1.0%), new cerebral infarction (2 patients, 2.0%) and new microbleeds (3 patients, 3.1%) (Table 3; Fig. 2, Fig. 3). Apart from these five, 2 additional patients had a new WMH, without a change in Fazekas score. After 3 months, the incidental DWI-positive lesions identified in the patient at baseline had converted to a WMH.

**Table 3:** Cerebrovascular MRI markers in COVID-19 patients on 3-month follow-up

	COVID-19 patients Follow-up MRI
Total number of patients (n)	98
Patients with new MRI markers, n (%)	5 (5.1%)
Time between baseline and follow-up MRI, days (median [IQR])	105.0 [92.0, 119.0]
Incidental DWI-positive lesions, n (%)	1 (1.0)
New white matter hyperintensities (measured as increase of Fazekas score), n (%)	0 (0.0)
New cerebral infarction, n (%)	2 (2.0)
New delayed hypoxemia, n (%)	0 (0.0)
New cerebral hemorrhage, n (%)	0 (0.0)
New microbleeds, n (%)	3 (3.1)
*Count, n (%)*	
1–10	3 (100.0)
>10	0 (0.0)
*Location, n (%)*	
Lobar	3 (100.0)
Deep	0 (0.0)
Cerebellar	0 (0.0)
Lobar and deep	0 (0.0)
Lobar and cerebellar	0 (0.0)
Lobar, deep and cerebellar	0 (0.0)

Abbreviations: COVID-19 = Coronavirus Disease 2019, DWI = diffusion-weighted imaging

After correction for potential confounders, hospitalization with COVID-19 was associated with a higher incidence of WMH (OR, 2.95 [95% CI: 1.07–8.57]) (Table 4). No association with COVID-19 was found for other MRI markers. Due to the low number of events of incidental DWI-positive lesions at baseline MRI and thus limited reliability in multivariable analysis, we only performed univariable analysis for incidental DWI-positive lesions.

**Table 4:** Association of COVID-19 with MRI markers at baseline corrected for confounders using multivariable logistic regression

MRI markers of cerebrovascular disease at baseline	Number of events (%)		Univariable analysis		Multivariable analysis	
	COVID+ *n* = 125	Controls *n* = 47 (reference)	OR (95% CI)	*P-value*	OR (95% CI)	*P-value*
DWI-positive lesions	1 (0.8)	1 (2.1)	0.37 (0.01–9.51)	0.486	N/A	***N/A***
White Matter Hyperintensities (any)	97 (77.6)	29 (61.7)	2.15 (1.04–4.43)	**0.038**	2.72 (1.04–7.41)	**0.044**
Microbleeds	29 (23.2)	6 (12.8)	2.06 (0.84–5.84)	0.136	2.20 (0.81–6.81)	0.143
Cerebral hemorrhage	6/ (4.8)	3 (6.4)	0.74 (0.19–3.62)	0.679	0.67 (0.09–5.54)	0.701

Abbreviations: COVID-19 = Coronavirus Disease 2019, DWI = diffusion-weighted imaging

## Discussion

In this study, we found no difference in prevalence of silent cerebral ischemia and other cerebrovascular MRI markers in unselected, hospitalized COVID-19 patients compared to healthy controls (with proven absence of previous SARS-CoV-2 infection without hospitalization) at baseline, apart from a higher burden of WMH. The prevalence of these markers in ICU vs. non-ICU patients was similar. After three months, 5.1% of the patients with COVID-19 had new brain MRI markers of cerebrovascular origin including incidental DWI-positive lesions, cerebral infarction and microbleeds.

We did not expect to find a comparable prevalence of silent cerebral ischemia in both patients and controls, considering that previous studies reported an increased risk of ischemic stroke in patients with COVID-19 [1, 4–8]. There are several possible explanations for this discrepancy. First, the pathophysiological mechanism of clinically overt ‘COVID-19-associated’ stroke may differ from silent ischemia. Although both types share risk factors, covid-associated stroke is often caused by a large vessel occlusion while silent ischemia is often associated with small vessel disease [21–24]. Second, patients in our cohort were enrolled after the first two waves (with two dominant variants: alfa b.1.1.7 & delta b.1.617.2) of COVID-19 in the Netherlands. After the first two waves the therapeutic guidelines had changed, and therefore the majority of our patients were treated with either low-molecular weight heparin, antiplatelet therapy or anticoagulation, which could have reduced thrombo-embolic complications [25]. Treatment with dexamethasone and tocilizumab was also introduced and large-scale vaccination campaigns were made available to the general population which could have led to a lower disease burden than in the first two waves. Together with possibly less pathogenic variants this could have reduced the risk of both clinically overt and silent cerebral ischemia [26, 27]. Third, we enrolled a low number of critically ill patients. Patients who are critically ill and admitted to an ICU are more likely to develop cerebrovascular disease (including critical illness encephalopathy and delayed cerebral ischemia) than patients with a less severe disease course [5, 9, 10]. Fourth, new ischemic lesions with diffusion restriction usually convert to small vessel disease markers such as WMH after 2–3 weeks. Due to local regulations preventing us from scanning patients for research while still infectious, the median time between onset of symptoms and baseline MRI was 6 weeks. The median time from onset of COVID-19-related symptoms to a COVID-related stroke has been reported to be approximately two weeks [4]. Therefore, it is possible that some, but not all, cases of silent cerebral ischemia have been missed which could have led to an underestimation of the true prevalence of silent ischemia.

Previous COVID-19 studies reported an increased prevalence of other cerebrovascular brain MRI markers (i.e. incidence of cerebral microbleeds up to 58.8%) on MRI in patients during the acute phase [14, 15, 28, 29]. Also, several studies showed an even higher prevalence of microbleeds (up to 71%) in patients admitted to an ICU compared to patients on the general ward [12, 28–30]. These findings are not in line with the results of our study, as we observed no difference in MRI markers between patients in the ICU and those in the general ward. A possible explanation might be the fact that patients selected in these studies underwent neuroimaging because of retrospective selection on neurological symptoms [31]. In CORONIS, all included patients were scanned regardless of neurological symptoms during admission. This gave us an insight in the prevalence of MRI markers in a regular and more generalizable population of hospitalized patients with COVID-19.

The prevalence of WMH in our study population is, with a mean age of 58 years, relatively high (77.6%), which may be explained by the high burden of vascular risk factors. Previous studies in the general population, with less vascular risk factors, described a prevalence ranging from 60% up to 90%, but these studies were mainly conducted in older patients (above 60) [32, 33]. One previous study that investigated adults between 50 and 59 years old reported a prevalence of 35.3% [34]. It is known that patients with cardiovascular risk factors have a higher risk at admission due to diseases as COVID-19 [35]. These risk factors are also associated with WMH and the association of COVID-19 with WMH could therefore be explained by such confounding factors. For important and well-known risk factors, we could adjust in the multivariable analysis, but additional and even unknown risk factors could have been missed and not been accounted for. Nevertheless, extensive WMH and signs of cerebral small vessel disease can be associated with cognitive, mental and physical function and these patients might be at risk of experiencing subsequent cognitive complaints described in patients with post COVID-19 condition [36–38].

During follow-up, in five patients new clinically silent MRI markers of cerebrovascular disease were found, including two patients with (silent) cerebral infarction (with in one of them also multiple incidental DWI-positive-lesions). One of these patients had pulmonary embolism during hospitalization, which could imply a hypercoagulable state. This patient developed silent ischemic lesions whilst receiving anticoagulation therapy for three months after discharge. The other patient reported no clinical symptoms and had an extensive cardiovascular medical history. An exact etiology for these brain abnormalities remains undetermined, since these infarcts were asymptomatic and therefore not investigated. The three patients with new microbleeds during follow-up already had prevalent microbleeds. Apart from the underlying cardiovascular risk factors which all of these patients had, it could be hypothesized that COVID-19 could have additionally triggered an ongoing prothrombotic state after the acute phase (including endothelial dysfunction) leading to these signs of cerebrovascular disease. This might persist for several weeks or even months after resolution of the infection, but this warrants further investigation [39–41]. A prior study showed a 90-day cumulative incidence of arterial thrombosis (including myocardial infarction and ischemic stroke) in hospitalized patients with COVID-19 of 3%, which is slightly lower than the incidence we found in our study [42]. These silent ischemic lesions could occur even more often than clinically overt ischemic stroke but due to their lack of symptoms they evade detection [43]. The presence of brain abnormalities in post-COVID patients has been associated with a lower risk of good recovery, so correlating clinically silent lesions to impaired outcome after COVID-19 could be a worthwhile pathophysiological target for research on these long remaining symptoms [44, 45].

To our knowledge, this is the first study to prospectively perform brain MRI in hospitalized COVID-19 patients, who were not exhibiting neurological symptoms. Strengths of our study included that patients were largely unselected, which reflects a comprehensive representation of the general hospitalized COVID-19 population in the Netherlands. Second, healthy controls were recruited throughout patients’ relatives and acquaintances, generating groups comparable in societal and environmental factors. Third, we repeated MRI after 3 months, which enabled us to investigate a possible ongoing disease process due to COVID-19.

Some limitations need to be considered. First, the median time between symptom onset and MRI was approximately 6 weeks, which could have led to an underestimation of silent ischemia. Second, as patients needed to provide written informed consent, the most critically ill patients may have been underrepresented, who either died during hospitalization, refused or were otherwise unable to provide written consent. Third, by using the Fazekas score as a qualitative assessment scale of white matter hyperintensities, it is possible that subtle changes in white matter hyperintensity volumes within patients may have been missed. Fourth, the relatively small sample size may have contributed to the inability to detect a significant difference resulting in seemingly neutral outcomes. Still, as far as we know, this is the largest sample size in an unselected prospective cohort study on MRI outcomes in hospitalized patients compared to controls. Fifth, constraint of the MRI budget has led to enrollment of a limited number of participants without previous SARS-CoV-2 infection without a formal pre-specified sample size calculation. The main focus of our study was on cerebrovascular abnormalities in patients with COVID-19, therefore we prioritized enrolling and scanning this group. The exploratory nature of our study means the precision of our prevalence estimates is limited and may affect the generalizability of our findings. Finally, a possible limitation is our inability to distinguish between the effects of COVID-19 and those attributed to the hospital admission itself. Our research question was to assess the impact of a severe COVID-19 infection, inherently linked to hospitalization—a clinical question. Addressing the more pathophysiological focus, regarding specifically the effects of COVID-19, would have required individuals hospitalized with a severe non-COVID illness undergoing MRI solely for research purposes, a prospect that raises ethical concerns but could be explored in a future study. However, our comparison with a healthy control group, where the contrasts were most pronounced and significant differences were not observed besides WMH, suggests that it is unlikely that including such a control group (hospitalized non-COVID patients) would have given any new insights.

### Conclusion

In this prospective multicenter cohort study of unselected hospitalized COVID-19 patients, we found overall no higher prevalence of cerebrovascular MRI markers, apart from WMH. The few incident DWI-lesions were most likely to be explained by well-known risk-factors for progression of small vessel disease. These findings suggest that severe COVID-19 infection has limited effects on cerebral small vessel disease in the general hospitalized patient without overt neurological symptoms.

### Electronic supplementary material

Below is the link to the electronic supplementary material.

Supplementary Material 1

## Data Availability

Data are available upon reasonable request. Requests may be sent to the corresponding author.
