# Supplementary material for: Prevalence and 3-month follow-up of cerebrovascular MRI markers in hospitalized COVID-19 patients: the CORONIS study
Source: Neuroradiology. 2024 Jul 2;66(9):1565–75. doi: 10.1007/s00234-024-03411-1 (PMC11322373; doi:10.1007/s00234-024-03411-1)
Supplement: Supplementary file 1 — Supplementary Material 1 [file 234_2024_3411_MOESM1_ESM.docx]

# **Supplemental material to:**

**Prevalence and 3-month follow-up of cerebrovascular MRI markers in hospitalized COVID-19 patients: the CORONIS study**

| **Overview Supplemental Material:**   - Supplemental Tables 1-4 - STROBE-guidelines manuscript |
| --- |

***Supplemental Tables***

**Supplemental Table 1 – MRI scanning protocol per center**

|  |  | Participating center | | |
| --- | --- | --- | --- | --- |
|  |  | Radboudumc | LUMC | UMCU |
| **Patients (n=)** |  | 64 | 45 | 16 |
| **Controls (n=)** |  | 26 | 17 | 4 |
| **Type of scanner** |  | Siemens 3T Prisma | Philips 3T Ingenia | Ingenia Elition 3T X |
| **Contrast agent** |  | 15 ml Dotarem® (0.5 mmol/mL) | Clariscan (0.2 ml/kg) | 0,1 ml Gadovist/kg |
| **Duration (in min)** |  | 40 | 35 | 25 |
| **T1-weighted** | Orientation | 3D | 3D | 3D |
|  | Voxels size | 0.9 mm isotropic | 1.15 mm isotropic | 0.5 mm isotropic |
| **FLAIR** | Orientation | 3D | Axial | Axial |
|  | Voxel size | 1 mm isotropic | 0.7 x 0.7 x 5.00 mm | 0.6 x 0.6 x 4 mm |
| **Diffusion weighted imaging (DWI)** | Orientation | Axial | Axial | Axial |
|  | Slice thickness + resolution | 5 mm | 5 mm | 4 mm |
| **Susceptibility weighted imaging (SWI)** | Orientation | Axial | 3D | Axial |
|  | Slice thickness + resolution | 3 mm | 2 mm | 2 mm |
| **Intracranial vessel wall imaging with and without contrast** | Orientation | 3D | 3D | 3D |
|  | Voxel size | 0.9 mm isotropic | 0.6 x 0.6 x 1.0 mm | 0.5 mm isotropic |
| **Diffusion tensor image (DTI)** | Size and b-value | 2mm isotropic,  B0, 1000, 2000  64 directions | - | - |

Abbreviations: Radboudumc = Radboud University Medical Center, LUMC = Leiden University Medical Center; UMCU= University Medical Center Utrecht, FLAIR = Fluid-attenuated inversion recovery

**Supplemental Table 2 - MRI markers of interest**

| MRI-sequence: | Outcome: | Additional information: |
| --- | --- | --- |
| FLAIR | White matter hyperintensities (WMH) | Fazekas (0/1/2/3) |
|  | Previous cerebral infarction | Location:   - Local, multifocal - Cortical, lacunar |
|  | Signs of delayed cerebral hypoxia |  |
| DWI | Acute ischemic lesions, incidental DWI-positive lesions | Location:   - Local, multifocal - Lacunar, territorial |
| SWI | Cerebral hemorrhage | Location |
|  | Cerebral microbleeds | Location: lobar, deep  Number of lesions: 1-10, >10 |
| Coincidental findings | Presence / absence |  |

Abbreviations: MRI = magnetic resonance imaging, FLAIR = Fluid-attenuated inversion recovery, DWI = Diffusion weighted imaging, SWI = susceptibility weighted imaging.

**Supplemental Table 3 - Clinical characteristics of the patients with COVID-19**

|  | COVID-19 patients (n=125) |
| --- | --- |
| Time of hospitalization, days (median [IQR]) | 8.0 [5.0-12.0] |
| Time between positive PCR and inclusion, days (median [IQR]) | 16.0 [5.0-12.0] |
| ICU admission, n (%) | 27 (21.6) |
| Confirmation of COVID-19 on imaging (X-ray, CT) , n (%) | 97 (85.1) |
| Oxygen therapy required during hospital stay |  |
| Oxygen suppletion therapy: Nasal cannula/non rebreathing mask, n (%) | 81 (64.8) |
| Non-invasive ventilation: Optiflow , n (%) | 24 (19.2) |
| Invasive ventilation: Intubation, n (%) | 15 (12.0) |
| Laboratory tests |  |
| Hemoglobin, median [IQR] × 10⁹/L | 8.7 [8.2-9.2] |
| Platelet count, median [IQR]× 10⁹/L | 203.0 [160.0-262.0] |
| INR, median [IQR] × 10⁹/L | 1.1 [1.0-1.2] |
| Neutrophils, median [IQR] × 10⁹/L | 5.1 [3.6-7.3] |
| Leukocytes, median [IQR] × 10⁹/L | 6.6 [4.9-8.8] |
| CRP, median [IQR] × 10⁹/L | 102.2 [54.0-154.8] |
| D-dimer, median [IQR] × 10⁹/L | 973 [535.0-1562.0] |
| Treatment during hospital admission |  |
| Prophylactic anticoagulation, n (%) | 117 (93.6) |
| Corticosteroids (dexamethasone), n (%) | 121 (96.8) |
| Antibiotics, n (%) | 59 (47.2) |
| Antiviral therapy (remdesivir, tenofovir, valaciclovir), n (%) | 5 (4.0) |
| Experimental/immunosuppressive therapy: | 43 (34.4) |
| *Anakinra, n (%)* | 2 (1.6) |
| *Casirivimab/imdevimab (REGEN-COV), n (%)* | 15 (12.0) |
| *Sarilumab, n (%)* | 25 (20.0) |
| *Sotrivomab, n (%)* | 1 (0.8) |
| Complications during hospital stay |  |
| Pulmonary embolism, n (%) | 22 (17.6) |
| Deep venous thrombosis, n (%) | 1 (0.8) |
| Bleeding, n (%) | 5 (4.0) |
| Blood transfusion, n (%) | 3 (2.4) |
| Rhythm disorder, n (%) | 5 (4.0) |
| (Proven) bacterial infection, n (%) | 27 (21.6) |
| Delirium, n (%) | 13 (10.4) |
| Seizures, n (%) | 0 (0.0) |
| Liver function abnormalities, n (%) | 24 (19.2) |
| COVID-19+ antibodies (tested in 60 patients) , n (%) | 32 (53.3) |
| Mortality, n (%) | 0 (0.0) |

**Supplemental Table 4 – Cerebrovascular MRI markers in ICU vs. general ward patients**

|  | ICU | Non-ICU | P-value |
| --- | --- | --- | --- |
| Total number of patients (n) | 27 (100.0) | 98 (100.0) | NA |
| Female, n (%) | 7 (25.9) | 43 (43.9) | 0.092 |
| Age at inclusion, years (median [IQR]) | 59.00 [53.00, 65.50] | 60.50 [49.25, 68.00] | 0.862 |
| White matter hyperintensities score | 22 (81.5) | 75 (76.5) | 0.585 |
| Fazekas score |  |  | 0.459 |
| Fazekas 0, n (%) | 5 (18.5) | 23 (23.5) |  |
| Fazekas 1, n (%) | 19 (70.4) | 55 (56.1) |  |
| Fazekas 2, n (%) | 3 (11.1) | 15 (15.3) |  |
| Fazekas 3, n (%) | 0 (0.0) | 5 (5.1) |  |
| Previous cerebral infarction, n (%) | 3 (11.1) | 15 (15.3) | 0.582 |
| Delayed hypoxemia, n (%) | 1 (3.7) | 0 (0.0) | 0.056 |
| Incidental DWI-positive lesions, n (%) | 0 (0.0) | 1 (1.0) | 0.598 |
| Cerebral hemorrhage, n (%) | 2 (7.4) | 4 (4.1) | 0.474 |
| Microbleeds, n (%) | 10 (37.0) | 19 (19.4) | ***0.054*** |
| *Location* |  |  | 0.144 |
| Lobar, n (%) | 3 (30.0) | 11 (57.9) |  |
| Deep, n (%) | 2 (20.0) | 4 (21.1) |  |
| Cerebellar, n (%) | 1 (10.0) | 1 (5.3) |  |
| Lobar and deep, n (%) | 3 (30.0) | 0 (0.0) |  |
| Lobar and cerebellar, n (%) | 1 (10.0) | 1 (5.3) |  |
| Lobar, deep and cerebellar, n (%) | 0 (0.0) | 2 (10.5) |  |
| *Count* |  |  | 0.149 |
| 0, n (%) | 17 (63.0) | 79 (80.6) |  |
| 1-10, n (%) | 8 (29.6) | 16 (16.3) |  |
| >10, n (%) | 2 (7.4) | 3 (3.1 |  |

***STROBE Statement—checklist of items that should be included in reports of observational studies – CORONIS study***

|  | Item No | Recommendation | Page  No |
| --- | --- | --- | --- |
| **Title and abstract** | 1 | (*a*) Indicate the study’s design with a commonly used term in the title or the abstract | 1 |
|  |  | (*b*) Provide in the abstract an informative and balanced summary of what was done and what was found | Abstract file |
| Introduction | | | |
| Background/rationale | 2 | Explain the scientific background and rationale for the investigation being reported | 2,3 |
| Objectives | 3 | State specific objectives, including any prespecified hypotheses | 2,3 |
| Methods | | | |
| Study design | 4 | Present key elements of study design early in the paper | 4 |
| Setting | 5 | Describe the setting, locations, and relevant dates, including periods of recruitment, exposure, follow-up, and data collection | 4 |
| Participants | 6 | (*a*) *Cohort study*—Give the eligibility criteria, and the sources and methods of selection of participants. Describe methods of follow-up  *Case-control study*—Give the eligibility criteria, and the sources and methods of case ascertainment and control selection. Give the rationale for the choice of cases and controls  *Cross-sectional study*—Give the eligibility criteria, and the sources and methods of selection of participants | 4 |
|  |  | (*b*) *Cohort study*—For matched studies, give matching criteria and number of exposed and unexposed  *Case-control study*—For matched studies, give matching criteria and the number of controls per case | 4 |
| Variables | 7 | Clearly define all outcomes, exposures, predictors, potential confounders, and effect modifiers. Give diagnostic criteria, if applicable | 4-7 + (Supplemental material, Tables S1+S2) |
| Data sources/ measurement | 8* | For each variable of interest, give sources of data and details of methods of assessment (measurement). Describe comparability of assessment methods if there is more than one group | 4-7 + (Supplemental material page, Tables S1+S2) |
| Bias | 9 | Describe any efforts to address potential sources of bias | 4 (referral protocol paper) |
| Study size | 10 | Explain how the study size was arrived at | 6-7 |
| Quantitative variables | 11 | Explain how quantitative variables were handled in the analyses. If applicable, describe which groupings were chosen and why | 5-6 |
| Statistical methods | 12 | (*a*) Describe all statistical methods, including those used to control for confounding | 6-7 |
|  |  | (*b*) Describe any methods used to examine subgroups and interactions | 6-7 |
|  |  | (*c*) Explain how missing data were addressed | 6-7 |
|  |  | (*d*) *Cohort study*—If applicable, explain how loss to follow-up was addressed  *Case-control study*—If applicable, explain how matching of cases and controls was addressed  *Cross-sectional study*—If applicable, describe analytical methods taking account of sampling strategy | 6-7 |
|  |  | (*e*) Describe any sensitivity analyses | 6-7 |

Continued on next page

| Results | | | |
| --- | --- | --- | --- |
| Participants | 13* | (a) Report numbers of individuals at each stage of study—eg numbers potentially eligible, examined for eligibility, confirmed eligible, included in the study, completing follow-up, and analysed | 8 , Figure 1 |
|  |  | (b) Give reasons for non-participation at each stage | 8 , Figure 1 |
|  |  | (c) Consider use of a flow diagram | 8 , Figure 1 |
| Descriptive data | 14* | (a) Give characteristics of study participants (eg demographic, clinical, social) and information on exposures and potential confounders | 8, Supplemental material, Table 3) |
|  |  | (b) Indicate number of participants with missing data for each variable of interest | 8,Supplemental material, Table 3) |
|  |  | (c) *Cohort study*—Summarise follow-up time (eg, average and total amount) | 8 (Table 3) |
| Outcome data | 15* | *Cohort study*—Report numbers of outcome events or summary measures over time | 9 + 10, table 2-4 |
|  |  | *Case-control study—*Report numbers in each exposure category, or summary measures of exposure | *N/A* |
|  |  | *Cross-sectional study—*Report numbers of outcome events or summary measures | 9+10, table 2 |
| Main results | 16 | (*a*) Give unadjusted estimates and, if applicable, confounder-adjusted estimates and their precision (eg, 95% confidence interval). Make clear which confounders were adjusted for and why they were included | 9+10 |
|  |  | (*b*) Report category boundaries when continuous variables were categorized | N/A |
|  |  | (*c*) If relevant, consider translating estimates of relative risk into absolute risk for a meaningful time period | N/A |
| Other analyses | 17 | Report other analyses done—eg analyses of subgroups and interactions, and sensitivity analyses | Supplemental material – Table 4) |
| Discussion | | | |
| Key results | 18 | Summarise key results with reference to study objectives | 12-14 |
| Limitations | 19 | Discuss limitations of the study, taking into account sources of potential bias or imprecision. Discuss both direction and magnitude of any potential bias | 14,15 |
| Interpretation | 20 | Give a cautious overall interpretation of results considering objectives, limitations, multiplicity of analyses, results from similar studies, and other relevant evidence | 12-16 |
| Generalisability | 21 | Discuss the generalisability (external validity) of the study results | 15-16 |
| Other information | | | |
| Funding | 22 | Give the source of funding and the role of the funders for the present study and, if applicable, for the original study on which the present article is based | Title Page |

*Give information separately for cases and controls in case-control studies and, if applicable, for exposed and unexposed groups in cohort and cross-sectional studies.

**Note:** An Explanation and Elaboration article discusses each checklist item and gives methodological background and published examples of transparent reporting. The STROBE checklist is best used in conjunction with this article (freely available on the Web sites of PLoS Medicine at http://www.plosmedicine.org/, Annals of Internal Medicine at http://www.annals.org/, and Epidemiology at http://www.epidem.com/). Information on the STROBE Initiative is available at www.strobe-statement.org.
